# Epidemiologic Methods Lessons Learned from Environmental Public Health Disasters: Chernobyl, the World Trade Center, Bhopal, and Graniteville, South Carolina 

**DOI:** 10.3390/ijerph9082894

**Published:** 2012-08-16

**Authors:** Erik R. Svendsen, Jennifer R. Runkle, Venkata Ramana Dhara, Shao Lin, Marina Naboka, Timothy A. Mousseau, Charles Bennett

**Affiliations:** 1 Department of Global Environmental Health Sciences, Tulane University School of Public Health and Tropical Medicine, Tulane University Health Sciences Center, ENHS SL-29, 1440 Canal St., Suite 2100, New Orleans, LA 70112, USA; 2 Department of Epidemiology and Biostatistics, University of South Carolina, Columbia, SC 29208, USA; Email: jennifer.r.runkle@emory.edu; 3 Nell Hodgson Woodruff School of Nursing, Emory University, Atlanta, GA 62234, USA; 4 Department of Environmental Health, Emory University, Atlanta, GA 62234, USA; Email: v.ramana.dhara@emory.edu; 5 Bureau of Occupational and Environmental Epidemiology, State University of New York-Albany, Albany, NY 84870, USA; Email: sxl05@health.state.ny.us; 6 Radioecological Center of the National Academy of Sciences, Kiev 04050, Ukraine; Email: marinavn1@yahoo.com; 7 Department of Biological Sciences, University of South Carolina, Columbia, SC 29208, USA; Email: mousseau@mailbox.sc.edu; 8 College of Pharmacy, University of South Carolina, Columbia, SC 29208, USA; Email: charlesleebennett@gmail.com

**Keywords:** environmental health, epidemiology, accidents and injuries, chemical safety, occupational health

## Abstract

*Background*: Environmental public health disasters involving hazardous contaminants may have devastating effects. While much is known about their immediate devastation, far less is known about long-term impacts of these disasters. Extensive latent and chronic long-term public health effects may occur. Careful evaluation of contaminant exposures and long-term health outcomes within the constraints imposed by limited financial resources is essential. *Methods*: Here, we review epidemiologic methods lessons learned from conducting long-term evaluations of four environmental public health disasters involving hazardous contaminants at Chernobyl, the World Trade Center, Bhopal, and Graniteville (South Carolina, USA). *Findings*: We found several lessons learned which have direct implications for the on-going disaster recovery work following the Fukushima radiation disaster or for future disasters. Interpretation: These lessons should prove useful in understanding and mitigating latent health effects that may result from the nuclear reactor accident in Japan or future environmental public health disasters.

## 1. Introduction and Methods

Despite our preparedness efforts, disasters are unpredictable, unannounced and unexpected. Occasionally disasters result in the release of a toxicological hazard into the affected environment. Such events may be considered environmental public health disasters (EPHD). Disasters are chronicled by stories of acute devastation and initial large-scale humanitarian relief efforts. Shortly thereafter, attention wanes and humanitarian efforts dwindle, while adverse long-term health effects may follow. Such effects are frequently poorly understood and even less so concerning long-term repeated exposures to a contaminated environment resulting from a disaster. Epidemiologic studies of EPHDs resulting from accidental or intentional releases of hazardous contaminants provide important information on long-term toxic health effects [1,2,3,4], though methodological challenges such as mitigating selection biases, exposure assessment, and community mistrust present important obstacles to comprehensively evaluating these long-term health effects. The purpose of this paper is to share some practical lessons learned during the public health recovery work after four EPHDs (Table 1). 

**Table 1:** Epidemiologic methods lessons learned from four Environmental Public Health Disasters.

Disaster	Location	Year	Population Scale	Exposure	Outcomes	Lessons Learned
Bhopal	India	1984	Hundreds of Thousands	Methyl isocyanate	Multiple systems	Sometimes simple studies are sufficient.
Chernobyl	Ukraine	1986	Hundreds of Thousands	Radioactive fallout	Multiple systems	Sometimes public health interventions can facilitate longitudinal studies. Sometimes “ecological epidemiology” can be a useful alternative to human studies.
World Trade Center Collapse	New York City	2001	Thousands	Caustic dusts	Mostly pulmonary	Sometimes careful inclusion of an unaffected control population is better than comparing distance to exposure only.
Graniteville	South Carolina	2005	Many hundreds	Chlorine gas (acidic)	Mostly pulmonary	Sometimes using longitudinal occupational health cohorts can augment the population-based studies.

It is our hope that these practical lessons will contribute to both current disaster recovery work, like with the Fukushima disaster, and the public health recovery work after future unfortunate disasters. We reviewed epidemiologic lessons learned from evaluations of four EPHDs in Europe, North America, and Asia which involved hazardous contaminants. Investigators directly involved with developing and implementing epidemiological studies following EPHDs in Bhopal, Chernobyl, New York City, and South Carolina collaboratively reviewed both their experiences and those of their collaborators, and discussed them collectively. The disasters in Bhopal and South Carolina illustrate impacts from exposure to a toxic gas while the World Trade Center (WTC) disaster in New York City demonstrates the effects of exposure to caustic dust particulates. The Chernobyl disaster illustrates impacts of a radiological accident involving emissions of aerosols and gasses with both acute and chronic impacts on surrounding regions. These lessons learned are not comprehensive accounts of the public health and epidemiological activities after these disasters. Rather, they are the accounts of scientists intricately involved in the research and recovery process with general discussions of the relevant issues illustrated within. 

## 2. Results

### 2.1. Bhopal

Lesson—sometimes simple studies are sufficient. In 1984 an industrial accident involving a 27 metric ton gas leak of methyl isocyanate (MIC) occurred at a pesticide plant in Bhopal, India [5]. This event affected over 500,000 people in nearby residential areas [6,7]. The magnitude of the accident, political and governmental inexperience in dealing with such a large-scale event, and the rapid dissolution of medical and social infrastructure impeded immediate evaluation of the toxic effects [8]. A decade later a transnational collaboration, the International Medical Commission on Bhopal (IMCB), was established to perform a cross-sectional epidemiologic study of the health effects of MIC exposure. To avoid self-selection bias, we performed simple random sampling for participant selection. Plume dispersion models (computer models using the available source, weather and topography data to predict where a chemical plume is dispersed within the atmosphere) were very imprecise due to the lack of sufficient local weather data. Therefore, exposure estimation was retrospectively assessed using three exposure indices: (1) physical activity during the event (e.g., fleeing gas *vs.* sheltering), (2) exposure duration, and (3) distance of residence from the pesticide plant. Current and past pulmonary symptoms were queried and an objective outcome assessment was performed: simple spirometry [7]. Hence, this study was susceptible to recall biases. Barriers to health care, traumatic stress, and disappointment with official policies were epidemic at the time of the study. It is commonly assumed that these issues may lead to inflated symptom reporting post-disaster. This study observed a decrease in both symptoms and objective spirometry deficits associated with increasing distance from the accident, suggesting that exposure misclassification from symptom over-reporting was likely non-differential [7] and not suggestive of recall bias. Though objective exposure measures were unobtainable the IMCB successfully executed a crude exposure assessment which suggested an association between MIC exposure and latent respiratory and pulmonary health effects [7]. The study showed that simple measures may be sufficient to link individual exposure estimates to observed health outcomes [6]. In most cases, a longitudinal study is the preferred method for assessing exposure and disease outcome among disaster victims over time. However, for Bhopal, the cross-sectional study circumvented issues of time, money, and changes in secular trends which can plague longitudinal cohort studies. Furthermore, a cross-sectional study can easily transition into a “cross-sectional cohort study” [9]; in which, a source population is sampled cross-sectionally and followed by retrospective assessments of participants” exposure and outcome histories over time. This type of study is ideal for chronic and recurrent diseases with low death rates, often associated with technological disasters [9]. For latent analysis of exposure-response, an individual exposure index based on duration of exposure, location, and physical activity is a relatively easy strategy to implement and can be used to validate the relationship between individual exposure and subjective and objective measures.

### 2.2. Chernobyl

Lesson—sometimes public health interventions can facilitate longitudinal studies. Soon after the Bhopal tragedy, in 1986 a nuclear reactor accident releasing radioactive isotopes including ^131^I, ^137^Cs, ^90^Sr and ^239^Pu occurred at the Chernobyl Nuclear Power Plant in the Ukraine. The accident was declared the worst nuclear power disaster in history, although the full impact of the Fukushima disaster remains unknown. Global plume dispersion models and aerial gamma-spectroscopy revealed high radioactive fallout in the Western Soviet Union, Eastern Europe, Western Europe, Northern Europe, and even North America, significantly contaminating more than 200,000 km^2^. Although impacts from the transient gaseous emissions of ^131^I on thyroid cancer rates have been well-documented [10,11,12], radiation exposure stemming from aerosolized radioactive heavy metals which is persistent in the soil due to long half-lives (30 and 29 years for ^137^Cs and ^90^Sr, respectively) has been less studied. Continuing transformation of plutonium to highly radioactive Am isotopes will persist for many years [13]. 

Early epidemiologic assessment of the Chernobyl disaster faced significant barriers. These included the impact of radionuclides moving through the food chain, lack of data among individuals who moved out of contaminated areas, variability in population-levels of radiosensitivity, missing data on the impact of individual radionuclides and their subsequent synergistic impact with other environmental hazards, and uncertainty in the consequences of nominal ionizing radiation doses and internal radiation exposures [14]. Methodological limitations included poor exposure reconstruction, inherent fluctuations in physical and chemical properties of radionculides, and inability to capture external and internal radiation levels of exposed populations. Furthermore, data collection failed to follow standardized scientific protocols in the rush to collect data within the immediate disaster period, resulting in questionable reliability. 

Soviet censorship and constraints related to historical data acquisition forced Ukrainian researchers to develop novel and cost-effective approaches to conducting epidemiologic assessments of Chernobyl. Bypassing the expense and time it would take to assemble a cohort, obtain detailed clinical and laboratory information, and accrue enough follow-up time to detect exposure-response associations, one approach harvested pediatric health data from existing Ukrainian public health surveillance systems. In 1986 the Soviets mandated that children age 0–14 years residing in the most contaminated and unevacuated areas of Russia, Belarussia, and Ukraine receive annual medical examinations. These were maintained by the Ukrainian government after the fall of the Soviet Union until 2006 when the funding for public health program was cut and the mandate halted. As a result, the Ukrainian Ministry of Health collected twenty years of longitudinal data on medical history, physical exams, and laboratory analyses for over 1 million children from the most contaminated regions of Ukraine. These data were collected for public health purposes with no initial plans to use them for scientific research. Transcription of only six years of such data from only one affected region has generated novel scientific findings when linked with exposure data and has been recently transitioned into an elective longitudinal cohort study [15,16,17,18]. Much more could be learned from these health records and the longitudinal cohort study that was born from them.

Due to dependence on subsistence farming in the most contaminated areas and the poor compliance with public health recommendations of avoiding local food sources, potential ionizing radiation exposure through ingestion of local food was a major public health concern. Therefore, public health officials tested the use of local soils to estimate personal radiation exposure. Soil measurements and the spatial distribution of radiation data were collected over several years (1987–1993). The Chernobyl Sasakawa Health and Medical Cooperation project provided evidence that individual ^137^Cs levels were highly correlated with residential soil contamination levels [19]. However, such soil exposure information was not readily available for research purposes until relatively recently.

To glean research from the available public health records, personal exposure estimates derived from residential soil contamination was linked to the public health screening data from the 6 years of abstracted data previously mentioned. This research used six-years (1993–1998) of Ukrainian Ministry of Health surveillance data representing ~75% of the children within one region (although the health screenings were mandated there was no enforcement). Many children born both before and after the 1986 accident were included in the cohort, allowing researchers to distinguish between environmental and in utero exposures. Results revealed that poor health outcomes among exposed children were associated with high levels of local soil ^137^Cs [16,20]. In response to these findings, public health officials quickly communicated the risks of local food consumption to at-risk regions yet again.

Lesson—sometimes “ecological epidemiology” can be a useful alternative to human studies. Because people are not the only organisms exposed, a second approach to evaluating the health impact of the Chernobyl disaster involved long-term ecological assessments of many sentinel species in areas with varying levels of radioactive contamination [21]. Groups used include birds, butterflies, dragonflies, grasshoppers, spiders, amphibians, reptiles, mammals and plants [22,23,24,25]. Such “ecological epidemiology” can be used to assess animal sentinels, or bioindicators of exposure, as estimates of potential human health effects and risks within the disaster region. Sensitivity analyses suggest that birds are particularly sensitive and easily measurable sentinels for the ecological effects of radionuclides in the environment [26] and recent comparisons with impacts on animals in Fukushima suggest that birds and butterflies are impacted both by acute radiotoxicity and chronic mutation accumulation effects [22,27]. These studies are not impacted by the traumatic stress effects of surviving a disaster or living within the context of disaster, nor how the resulting health behaviors may modify toxicological effects. Such potential counfounders require careful assessment in human disaster studies [28]. These sentinel animal studies are relatively cost-effective, out-migration is rare if studying a species with a small home range, and clinical, laboratory, and pathology studies can be conducted efficiently. As in the prior human epidemiology study [16], radiation exposure can be proxied by measurements of ionizing radiation in the soil. Now, over a quarter century after the accident, sentinel animal studies have indicated effects of ionizing radiation on brain size [29], tumors and other developmental abnormalities [30], decreased sperm motility and higher frequencies of defective sperm [31], and higher bacterial loads on birds who resided closer to the disaster site. Such studies provide empirical evidence that ionizing radiation likely has long-term sentinel health effects, not simply effects due to poor health behaviors or psychological stress [32].

### 2.3. The World Trade Center

Lesson—sometimes careful inclusion of an unaffected control population is better than comparing distance to exposure only. Many studies now document that exposure to extremely caustic dust from the collapse of the World Trade Center (WTC) has increased the long-term medical needs of victims, responders, and clean-up workers [33,34,35,36,37,38,39]. One set of studies evaluated changes in respiratory health from excessive dust exposure among residents living near the former WTC in New York City [35,40]. Methodological challenges to this assessment included identification of exposure-associated outcomes, exposure misclassification, recall and selection bias. Similar to the Bhopal disaster, direct exposure measurements were unavailable; therefore residential distance from the WTC was used as an exposure proxy. However, unlike the Bhopal disaster, the target population included people both affected and unaffected (control): 9,200 residences within 1.5 kilometers of the former WTC (the affected area) and 1,000 residences in upper Manhattan more than 9 kilometers from the site (control area). To minimize selection bias and improve response rates, participation was encouraged among individuals both with and without breathing problems. By comparing demographics between respondents and the overall population, exclusion criteria were instituted to minimize differential selection by study area. Recruitment was intensified in “target” areas to improve response rates and better evaluate participation bias. Response rates were similar in both the affected and control areas. Reporting bias was minimized by qualitatively and quantitatively asking symptomology questions incorporating questions on exact time periods, severity, and exposure frequency [40]. The expectation of exposure misclassification was attenuated by excluding participants with evidence of residential mobility and other exposures not related to their geographic location [40]. Area analyses showed higher elevated rates of respiratory diseases in both recruitment targeted and non-targeted areas directly impacted, reducing concerns about selection bias. Assessment of distance from the event successfully informed public health officials of the disaster’s health effects on neighboring communities.

Lesson—sometimes using longitudinal occupational health cohorts can augment the population-based studies. A second set of studies evaluated long-term consequences of exposure to WTC dust by characterizing prospective trends in pulmonary function for seven years following the disaster [41]. This study examined changes in forced expiratory volume in 1 second (FEV1) among Federal Department of New York (FDNY) rescue workers. This study used occupational health surveillance data, including before and after spirometric measurement, on 92% of the 13,954 FDNY personnel (11,868 firefighters and 2,086 EMS workers) present at the WTC site between 11 September and 24 September, 2001. A robust natural experiment design which contrasted before and after trends in lung function (*i.e.*, interrupted longitudinal cohort) determined little or no recovery of average lung function decline during the 6-year follow-up period. From 2002 through 2008, FEV1 values continued to decline. The overall loss in lung function from early 2001 until late 2008 averaged almost 600 mL for firefighters who had never smoked and more than 500 mL for Emergency Medical System (EMS) workers who had never smoked. FEV1 values declined at an average rate of 25 mL per year for firefighters who had never smoked and 40 mL per year for EMS workers who had never smoked. Though values are within the range of 20 to 56 mL per year reported for healthy, nonsmoking men between the ages of 24 and 65, the rate of decline among EMS workers was significantly faster than that for firefighters. This study demonstrated the utility of using longitudinal data from an occupational health surveillance system in disaster epidemiologic research.

### 2.4. Train Derailment in South Carolina

Lesson—sometimes community engagement in the public health and research activities mitigates selection biases. In 2005 a late-night train derailment adjacent to a textile mill in Graniteville, South Carolina released approximately 60 tons of chlorine gas in the town center [42]. State health and environmental officials partnered with the Centers for Disease Control and Prevention to immediately evaluate the health effects. Rapid epidemiologic assessment was initiated while advancing the public health agenda of targeting needed resources to the affected community [43]. Mandatory reporting of persons treated for chlorine-related symptoms accelerated active case finding. Emergency department logs from hospitals in South Carolina and Georgia revealed that acute pulmonary toxicity precipitated nine immediate fatalities and 71 hospitalizations immediately after the train derailment [44]. Though not all were hospitalized, we know of 840 who required medical care [28]. In order to evaluate the urgent long-term health needs following chlorine exposure, a community based participatory research project (CBPR) was initiated and is on-going [20]. This collaboration initially resulted in two public health interventions designed to assess the persistent health effects of those most likely affected and their need of medical assistance, including mental health assessment [28]. In order to evaluate the urgent long-term health needs after the chlorine exposure, the community was engaged within the public health planning and research process. This public health/community collaboration initially resulted in two public health interventions designed to assess the persistent health needs of those most likely affected and their need of medical assistance [45,46], including mental health assessment [28]. As the other disasters have previously illustrated, there is a constant struggle to mitigate selection bias in epidemiologic studies of disaster populations. This is even more of a concern when there is the likelihood of health disparities across race [47] and lack of trust in public officials who some may think should have prevented the EPHD. Distrust is a very normal response because trust has been broken in EPHDs, and such affected communities are often already socio-economically disadvantaged [48]. Therefore it is imperative to fully engage the study community within the scientific approach, building trust, and establishing local credibility prior to initiating any studies [49]. When a community is a partner in the scientific process with a vested interest in the results, they are more likely to participate and comply with the study protocol. We did so from the beginning in Graniteville with many town-hall meetings, outreach to churches and pastors, door-to-door campaigns, collaboration with the local schools and businesses, community information workshops, health fairs and picnics, *etc*. Community engagement successfully overcame the selection bias issue in the Graniteville public health interventions [20]. This community engagement and collaboration has fostered supplemental community based participatory research (CBPR) which is on-going [20]. CBPR maximizes participation rates and minimizes attrition by engaging the participants in the entire research process [49,50,51,52,53,54,55,56,57,58,59,60,61,62,63,64,65,66,67,68]. Therefore, CBPR strategies are crucial when studying a community impacted by an environmental disaster [69,70,71,72,73,74,75,76,77]. A longitudinal study using CBPR is currently evaluating pulmonary function among a cohort of Graniteville millworkers with baseline pulmonary function tests recorded prior to the chlorine spill. This millworker study will employ a natural experimental design comparing before and after lung health in an interrupted time series. Personal exposure estimates will be generated from plume dispersion modeling [78]. Key successes in evaluating this EPHD were joint consideration of public health needs and epidemiologic research methods, involving the community in short- and long-term assessments and supplemental CBPR, developing a computer model of the plume, leveraging occupational health surveillance data for public health purposes, and accessing existing medical record information to evaluate disease severity within the affected community.

## 3. Discussion

The effects of EPHDs are wide-ranging. Lessons learned from the public health evaluation of EPHDs involving contaminant dispersal at the World Trade Center, Bhopal, Chernobyl, and South Carolina provide insights about study design, exposure and outcome assessments, and have implications for evaluating long-term health effects of both recent (e.g., Fukushima) and future disasters.

EPHDs can include hazards with little documented human toxicity data. Therefore, any novel reports of health effects within such exposed populations advance our greater toxicological understanding. But performing epidemiological studies in EPHD populations is not easy. Groups representative of increasing exposure and a similar unexposed group from within the same population [79,80] should be included, even if only using self-reported exposure location data. Public health researchers must judiciously decide upon participant enrollment plans, operational definitions, inclusion/exclusion criteria, and appropriate study designs while minimizing harm and maximizing benefit. Appropriate participant selection, migration patterns, temporal sequence uncertainty, and exposure estimation are major challenges in EPHD studies. However, appropriate exposure assessment during both the early and late phases of an environmental disaster is vital. During the early phase, identification and quantification of released material and characterization of dispersion patterns helps define the at-risk population, determine potential health risks, and enable exposure estimation [81]. At the WTC and Bhopal, recall of exposure location alone successfully facilitated exposure estimation. During the late phase exposure assessments can further advise public health interventions, identify populations suitable for observational study, and evaluate exposure mitigation strategies. After Chernobyl, exposure measures for individuals were proxied by soil ^137^Cs measurements and used successfully in a new cohort study [16].

Epidemiological study of discrete communities with relatively localized hazardous exposures can produce significantly fruitful information about the toxicity of the exposure hazard. However, such studies require logistical care because of mistrust among the affected communities which have been violated could result in poor participation, high attrition, volunteer bias, and failed studies [5,82,83,84,85,86,87]. To directly address mistrust concerns, the WTC and Graniteville efforts used community based participatory research related principals (CBPR). In CBPR research investigators partner with the disaster population to identify research questions, plan, implement, evaluate, and disseminate targeted interventions. Scientists must choose to first focus on the public’s health needs and the science second when working within EPHDs.

Epidemiological investigations following EPHDs are uniquely poised to accomplish etiologic research [88]. As noted in these case studies, natural experiment designs (e.g., pre/post or interrupted time-series) can be useful when pre-disaster baseline data are available, as with pulmonary function among firemen and EMS workers at the WTC and millworkers in South Carolina. In the case of smaller scale events, statistical power is maximized by using longitudinal designs. When exposure is uniformly nested in a closed longitudinal cohort, each participant serves as their own continuously equivalent control. This design is robust to potential confounding effects of time-invariant covariates (e.g., sex, race), while adjustment may still be needed for time-variant covariates (e.g., season, interviewer). However, because this design assumes uniform and randomly distributed exposure within the study population, selection of appropriate exposure groups requires the same rigor called for in other observational study designs.

Longitudinal cohort studies can be used to determine incident health consequences from an EPHD. In the case of the Chernobyl disaster a longitudinal cohort study was not an initially feasible method for monitoring latent health effects from ionizing radiation exposure. Soviet secrecy was responsible for strategic censorship and data falsification [14]. Therefore, due to the difficulties in obtaining reliable data, there are relatively few conclusive research studies on the non-cancer health effects of ionizing radiation stemming from the Chernobyl disaster despite the many thousands who have been exposed.

Lastly, “ecological epidemiology” studies can provide important insights into hazard exposure *versus* non-hazard exposure causes of latent health effects after EPHDs [29]. The Chernobyl sentinel studies, for example, provide a cost-effective option for evaluating long-term complications and are a nice template for building the arguments for follow-up human health studies.

## 4. Conclusions

EPHDs studies involving dispersal of environmental hazards fill important toxicology knowledge gaps in the latent health effects of these agents. We found several lessons learned from our collective disaster epidemiology work in such EPHDs. These include that sometimes simple study designs are sufficient, public health interventions can facilitate longitudinal studies, “ecological epidemiology” can be a useful alternative to human studies, careful inclusion of an unaffected control population may be better than comparing distance to exposure only, using longitudinal occupational health cohorts can augment population-based studies, and community engagement in the public health and research activities may mitigate selection biases. Furthermore, protocols for immediate and long-term assessment of environmental exposures should be commissioned rapidly in initial response efforts. While difficult methodological challenges exist in health studies of disasters, they are not insurmountable. Discussions of methodological issues should take into consideration both good science and public health practices. Sound scientific research elucidates exposure and response associations, examines the competence of extant response measures, and informs intervention strategies that can be used to evaluate and potentially mitigate long-term EPHD complications.
